# Plant growth promotion and *Penicillium citrinum*

**DOI:** 10.1186/1471-2180-8-231

**Published:** 2008-12-22

**Authors:** Sumera Afzal Khan, Muhammad Hamayun, Hyeokjun Yoon, Ho-Youn Kim, Seok-Jong Suh, Seon-Kap Hwang, Jong-Myeong Kim, In-Jung Lee, Yeon-Sik Choo, Ung-Han Yoon, Won-Sik Kong, Byung-Moo Lee, Jong-Guk Kim

**Affiliations:** 1 Department of Life Science and Biotechnology, Kyungpook National University, South Korea; 2 Department of Agronomy, Kyungpook National University, South Korea; 3 Department of Biology, Kyungpook National University, South Korea; 4 Department of Agricultural Bio-resource, National Academy of Agricultural Science, RDA, South Korea; 5 Department of Herbal Crop Research, National Institute of Horiticultural & Herbal Science, RDA, South Korea; 6 Department of Horticulture, University of California-Davis, USA; 7 Center of Biotechnology, University of Peshawar, Pakistan

## Abstract

**Background:**

Endophytic fungi are known plant symbionts. They produce a variety of beneficial metabolites for plant growth and survival, as well as defend their hosts from attack of certain pathogens. Coastal dunes are nutrient deficient and offer harsh, saline environment for the existing flora and fauna. Endophytic fungi may play an important role in plant survival by enhancing nutrient uptake and producing growth-promoting metabolites such as gibberellins and auxins. We screened roots of *Ixeris repenes *(L.) A. Gray, a common dune plant, for the isolation of gibberellin secreting endophytic fungi.

**Results:**

We isolated 15 endophytic fungi from the roots of *Ixeris repenes *and screened them for growth promoting secondary metabolites. The fungal isolate IR-3-3 gave maximum plant growth when applied to waito-c rice and *Atriplex gemelinii *seedlings. Analysis of the culture filtrate of IR-3-3 showed the presence of physiologically active gibberellins, GA_1_, GA_3_, GA_4 _and GA_7 _(1.95 ng/ml, 3.83 ng/ml, 6.03 ng/ml and 2.35 ng/ml, respectively) along with other physiologically inactive GA_5_, GA_9_, GA_12_, GA_15_, GA_19_, GA_20 _and, GA_24_. The plant growth promotion and gibberellin producing capacity of IR-3-3 was much higher than the wild type *Gibberella fujikuroi*, which was taken as control during present study. GA_5_, a precursor of bioactive GA_3 _was reported for the first time in fungi. The fungal isolate IR-3-3 was identified as a new strain of *Penicillium citrinum *(named as *P. citrinum *KACC43900) through phylogenetic analysis of 18S rDNA sequence.

**Conclusion:**

Isolation of new strain of *Penicillium citrinum *from the sand dune flora is interesting as information on the presence of *Pencillium *species in coastal sand dunes is limited. The plant growth promoting ability of this fungal strain may help in conservation and revegetation of the rapidly eroding sand dune flora. *Penicillium citrinum *is already known for producing mycotoxin citrinin and cellulose digesting enzymes like cellulase and endoglucanase, as well as xylulase. Gibberellins producing ability of this fungus and the discovery about the presence of GA_5 _will open new aspects of research and investigations.

## Background

Gibberellins (GAs) is a family of diterpenoid plant hormones, first detected in 1930s from culture filtrates of *Gibberella fujikuroi*, a known pathogen of rice plants [1,2]. In 1956, GA was isolated and identified as plant hormone from the extracts of higher plants [3]. GA appear to be involved in every aspect of plant growth and development, but their most typical (and spectacular) property is the enhancement of stem growth [4]. GA may modify the sex expression of flowers, induce the parthenocarpic development of fruit and delay senescence. They obviate the need for exposure to red light in the germination of seeds and spores, and the need for vernalisation in the growth of bulbs and tubers. They are associated with the breaking of winter dormancy and stimulate the formation of hydrolytic enzymes in germinating cereal grain [5]. Currently 136 GAs have been identified, while 12 fungi, pathogenic and non-pathogenic, associated with plants and/or soil has been reported as GA producers [6,7].

Endophytic fungi have been found to colonize the roots of plants [8] and have usually been defined as those fungi growing asymptomatically within the tissues of their host plants, excluding pathogenic fungi and mycorrhizae [9,10]. This relationship is subjected to change and replacement by other endophytes depending on environmental conditions [11] and host requirement. Fungal endophytes of roots can therefore be defined as those fungi located within apparently healthy, functional root tissues at the moment of sample collection [8]. Unlike mycorrhizal fungi, endophytes reside entirely within host tissues and may emerge during host senescence [12]. Endophytes have been shown to confer fitness benefits to host plants including tolerance to herbivory, heat, salt, disease, and drought, and increased below- and above-ground biomass [8,13-17]. Endophytic colonization may also improve the ecological adaptability of the host by enhancing tolerance to biotic and abiotic stresses [11,18]. *Penicillium citrinum *has been reported as common endophytic fungus of cereal plants like wheat and soybean. It had been isolated from different environmental conditions, ranging from permafrost sediments to agricultural fields and forest soils. It is most vigorously studied laboratory taxon that gained importance after discovery of well-known mycotoxin citrinin. Currently it is being explored for different secondary metabolites it can produce and their associated benefits [19-23].

Sand dunes in coastal regions of South Korea are being destroyed due to anthropogenic disturbances such as military action as well as beach construction and recreational activities. The intensity of artificial activities had affected the speed and efficiency of their conservation and revegetation [24,25]. We investigated root endophytic fungi of sand-dune flora for GAs production as such work had not been carried out in the past. This paper report screening of *Ixeris repens*, a common plant of sandy beaches across East Asian countries [25,26] for plant growth promoting metabolites by root endophytic fungi.

## Results

### Screening for plant growth promoting activity of fungal metabolites on waito-c rice

**Figure 1:**
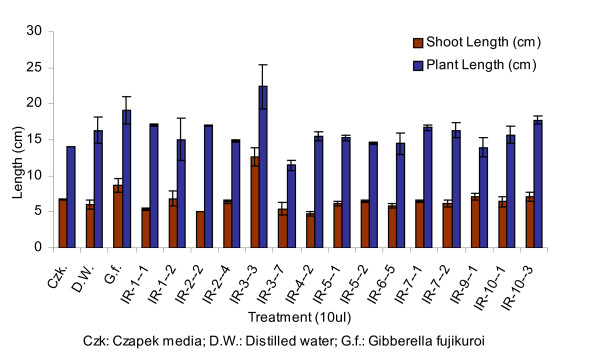
**Effect of fungal culture filtrates on length of waito-c rice seedlings after 7 days of incubation**. The experiment was conducted in 3 replicates; standard deviation from means was calculated using MS-EXCEL. Whole plant lengths as well as shoot lengths were increased after 7 days of treatment with culture filtrate. Culture filtrates of IR-3-3 caused shoot elongation more than that by *G fujikuroi *(Control III), but whole plant length was about same for both indicating little or no effect of filtrate on root elongation.

Fifteen endophytic fungi were isolated from roots of *Ixeris repens*, collected from sand dune at Pohang beach, Korean peninsula. The fungal culture filtrates were applied on waito-c rice seedlings and the length of seedlings was checked after one week of fungal culture filtrate application. Out of 15 isolated fungi, 2 fungal isolates considerably promoted shoot lengths of rice seedlings. The fungal isolate IR-3-3 gave maximum plant and shoot length of 22.35 cm and 12.60 cm respectively, while culture filtrate of IR-10-3 gave plant height of 17.75 cm and shoot length of 7.1 cm. The plant and shoot growth promotion by *G. fujikuroi *was much lower than IR-3-3, which was thus selected for further investigation (Figure 1).

### Bioassy of IR-3-3 for plant growth promoting activity on *Atriplex gemelinii*

**Figure 2:**
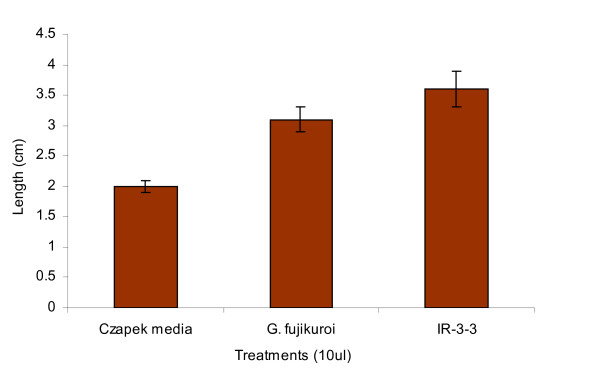
**Effect of IR-3-3 culture filtrate on *Atriplex gemelinii *seedlings after 15 days of application**. The experiment was performed in 3 replicates; standard deviation from means was calculated using MS-EXCEL. Shoot lengths of seedlings were increased after 15 days of culture filtrate treatment. IR-3-3 culture filtrate treated seedlings showed increased shoot lengths than those by wild type *G fujikuroi *indicating possibility of GAs production as secondary metabolite by the strain.

Since the growth rate of sand dune plants are much slower, shoot lengths of *Atriplex gemelinii *seedlings treated with IR-3-3 culture filtrate were measured after 15 days of culture filtrate application. The growth promoting capacity of IR-3-3 was compared with the results obtained from the application of culture filtrate of wild type *G. fujikuroi *and Czapek broth, separately. Since Czapek medium contain nutrients, its control was used to observe possibility of nutrients effect on shoot elongation. Shoot length of IR-3-3 culture filtrate treated seedlings was 3.6 cm, which was higher than that of *G. fujikuroi *culture filtrate (3.1 cm) and Czapek broth (2.0 cm) treated seedlings (Figure 2).

### Analyses of culture filtrate of IR-3-3 for the presence of gibberellins

**Figure 3:**
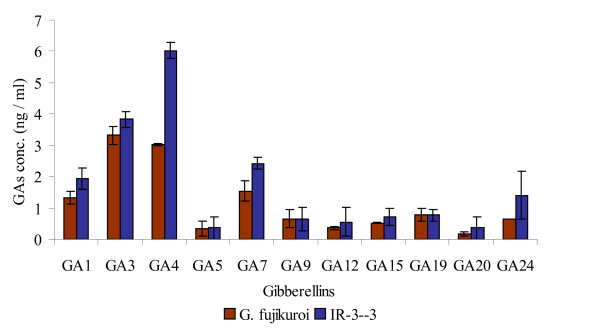
**Gibberellins content of fungal isolate IR-3-3 and wild type *G. fujikuroi***. The experiment was performed in 3 replicates; standard deviation from means was calculated using MS-EXCEL. On GC-MS SIM analysis of culture filtrate extracts from IR-3-3, all four bioactive GAs were detected higher than of *G. fujikuroi*. Non-bioactive GAs including GA_15_, GA_20 _and GA_24 _were in comparatively higher amounts, GA_9 _and GA_19 _were in about equal amounts, while GA_12 _was detected in amount lower than in case of control (*G. fujikuroi*).

Gibberellins analysis showed the presence of GA_1 _(1.95 ng/ml), GA_3_(3.83 ng/ml), GA_4 _(6.03 ng/ml), GA_5 _(0.365 ng/ml), GA_7 _(2.35 ng/ml), GA_9 _(0.65 ng/ml), GA_12 _(0.11 ng/ml), GA_15 _0.72 ng/ml), GA_19 _(0.67 ng/ml), GA_20 _(0.30 ng/ml) and GA_24 _(1.40 ng/ml) in culture filtrate of IR-3-3. Among them, GA_1_, GA_3_, GA_4 _and GA_7 _are physiologically active GAs (See Additional files 1, 2, 3, 4 for GC-MS SIM spectra of these GA). Fungal isolate IR-3-3 showed higher GAs productivity than wild type *G. fujikuroi *(see Figure 3). Important finding was the detection of GA_5 _in the culture filtrates of fungal isolate IR-3-3 and wild type *G. fujikuroi *(See Additional files 5 and 6 for GC-MS SIM spectra of this GA).

### Phylogenetic analysis

**Figure 4:**
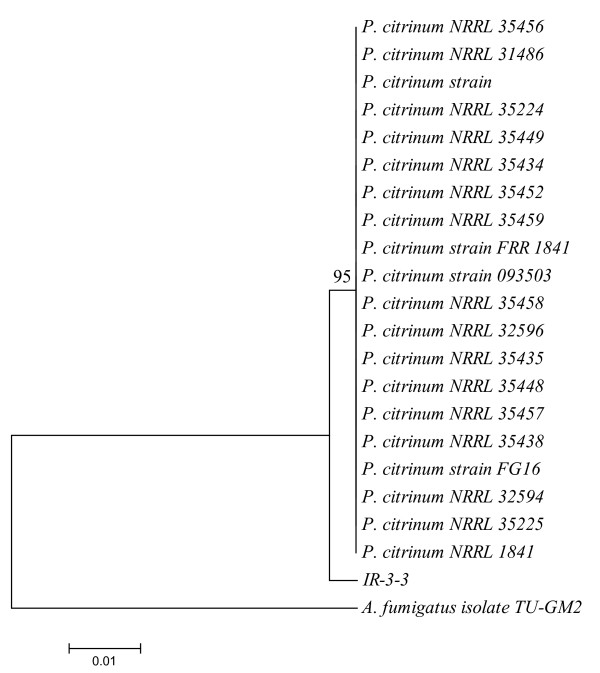
**Phylogenetic analysis of isolate IR-3-3**. A neighbor joining tree was constructed with 22 strains (21 strains of *P. citrinum *obtained through Blast, and one clone strain IR-3-3). *A. fumigatus *was used as outgroup for rooting the tree. Fungal isolate IR-3-3 formed 95% bootstrap support for monoclade formed by all members of *P. citrinum*, after 1000 bootstrap replications, strongly supporting its identification as a new strain of *P. citrinum*.

The phylogenetic analysis of fungal isolate IR-3-3 was carried out by distance tree construction. We aligned ITS1 sequences of available *P. citrinum *through BLAST sequence using ClustalW and a neighbour joining tree was constructed from 22 (21 references and 1 clone) aligned sequences. *A. fumigatus *was used as an out group for tree rooting. The fungal isolate IR-3-3 gave 95% bootstrap support for a monoclade of *P. citrinum *strains (1000 bootstrap replications), thus suggesting IR-3-3 as a new strain of *P. citrinum *(Figure 4).

On the basis of sequence homology and phylogenetic analysis, isolate IR-3-3 was thus identified as a new strain of *P. citrinum*. The ITS1 sequence of this strain has been submitted to GenBank database [GenBank: EU821333]. The fungal isolate was deposited to the Korean Agricultural Culture Collection (KACC) and was allotted no. KACC43900. The IR-3-3 was thus named as *P. citrinum *KACC43900.

## Discussion

Endophytic fungi form mutualistic interactions with their host, the relationship therefore being beneficial for both partners [8]. Mutualism frequently leads to enhanced growth of the host. In current study, the presence of plant growth promoting metabolites in culture filtrates of our fungal strains were determined through a primary screening experiment on waito-c rice seedlings. Use of rice seedlings is beneficial as they easily grow under controlled and sterilized conditions, hydroponically, using autoclaved water-agar media. Since this media is devoid of any nutrient, the sole effect of culture filtrate can easily be estimated. Waito-c rice is a known dwarf rice cultivar with reduced GA biosynthesis. Treatment of its seeds with uniconazol, a GA biosynthesis retardant, further suppresses the endogenous GAs production by blocking its biosynthesis pathway in the plant. Shoot elongation of these seedlings can be easily attributed to the activity of plant growth promoting secondary metabolites from fungal culture filtrates [27,28]. The microbial extracts had been and will continue to be a productive source of biologically active compounds [8]. Screening of microbial secondary metabolites is an established method for the identification of novel and biologically active molecules [29-32].

The plant growth promoting capacity of fungal secondary metabolites was confirmed by conducting a bioassay experiment on seedlings of *Atriplex gemelinii*, a commonly occurring plant of sandy shores in China, Japan and Korea [26]. Seeds of this plant did not exhibit dormancy, and germinate by soaking in water in a week time. Being dune-plant, it showed adaptation to nutrient deficiency, growing in hydroponic media (0.8% water-agar) without any supply of nutrient solution. Although germination is rapid in these plants, but they possess slow growth rate (0.5 m annually), therefore the seedling length was much lower than those of rice. However, considerable increase in its shoot lengths was observed, when treated with IR-3-3 as compared to control. Current findings confirmed the previous reports of shoot length promotion by fungal culture filtrate application [27,28].

GA analysis of the culture filtrates of IR-3-3 and *G. fujikuroi *showed that bioactive GA production capacity of IR-3-3 was much higher than wild type *G. fujikuroi*, which narrates the significance of this newly isolated fungal strain. The discovery of GA_5 _in the culture filtrates of both IR-3-3 and *G. fujikuroi *will lead to the modification of fungal GA biosynthesis pathway by providing the missing links for GA biosynthesis in fungi. Gibberellins were analyzed with gas chromatograph-mass spectrometer (GC-MS), which is a useful tool because of its ability to analyze highly complex mixtures and to detect compounds of different classes simultaneously. GC-MS techniques have been used in clinical medicine and were only recently introduced in plant research [30,33,34]. Recently, advances of mass spectrometry in food-related research [35] and aroma analysis [36] have been reported. Phytohormones quantification by GC-MS-SIM is well established and most reliable technique in ongoing investigations worldwide [27,28,37-39]. The major advantage of GC-MS is its unbiased character. In comparison with non-MS detection based chromatographic techniques (HPLC-DAD, GC-FID), where only compounds targeted by a special analytical protocol are found, GC-MS analysis can result in interesting and unexpected new knowledge about a particular extract [40]. Quantitative analysis is done by acquiring compound-specific molecular ions in selected ion monitoring (SIM) to increase the signal-to-noise ratio (SNR) of the MS experiment [41].

Approximately 70–80,000 species of fungi exists [42]. Due to possible existence of different morpho/biotypes of fungi within single species, traditional morphological and biochemical methods are not considered reliable for identification [31]. On the other hand, DNA sequence analysis methods are objective, reproducible and rapid means of identification, and thus gaining importance and have commonly been used to identify non sporulating endophytes [43-45]. We used 5.8S gene and flanking ITS1/4 regions for fungal identification. It is because highly conserved 5.8S gene is suitable for higher taxanomic level analysis while highly variable ITS regions are useful for analysis at lower taxanomic levels [42,46,47]. Constructing phylogenetic tree is crucial in molecular identification, since BLAST search alone cannot overcome possibilities of statistical errors. Bootstrap consensus is applied to the constructed tree so as to read maximum sequence replications. Neighbour joining tree with bootstrapping gave us a clear picture for identifying fungal isolate IR-3-3. It is because more than 100 BLAST hits belonged to *Penicillium *genera, thus strongly recommending our isolate as a member of this group. Although many phylogenetic trees exist and are used for analysis, neighbour-joining method has been designated most reliable tree construction method especially when dealing with closely related strains under varying rates of evolution [48,49].

## Conclusion

From our study we can conclude that root endophytic fungi play very important roles for supporting their host plants. Under environmental stress condition like that of coastal zones, waterlogged and barren lands with few plants, the endophytic fungi with extraordinary metabolites might be isolated and identified, and their metabolites can be used for human and environmental benefits. Further studies with *P. citrinum *KACC43900 regarding characterization of GAs encoding genes and optimization of GAs production media are suggested.

## Methods

### Plants, Fungal Strains, Culture Medium and Growth Conditions

Screening and isolation of plant root fungi was carried out on Hagem minimal medium plates supplemented with 80 ppm Streptomycin [50,51]. For storage, potato dextrose agar (PDA) plates and slants were used, while Czapek broth medium containing 1% glucose and peptone was used for Gibberellin production [52] by incubating strains at 30°C and 120 rpm for 7 days. The wild type strain of *Gibberella fujikuroi *provided by the Korean Culture Center of Microorganisms (KCCM) was used as a control during the experiment.

### Isolation of endophytic fungi from roots of *Ixeris repens*

The root samples were washed with tap water to remove sand particles and other debris, treated with Tween 80 solution and surface sterilized using perchloric acid (1%) solution. The surface sterilized roots were then cut into 0.5 cm pieces in laminar flow hood, cultured on Hagem media plates and, incubated at 25°C until emergence of fungi from inside of root pieces [53,54]. The isolated pure cultures of root fungi were stored on PDA plates and slants. Effectiveness of surface sterilization procedure was determined by imprinting sterilized root pieces on Hagem plates. Absence of any microbial growth on imprinted plates after 4–7 days of incubation were considered enough for effective surface sterilization of roots.

### Screening of fungal culture filtrate for plant growth promoting metabolites on rice

The culture filtrate of fungal isolate was bioassayd on waito-c rice seedlings for the presence of plant growth promoting metabolites. For this purpose, the fungal isolate was grown in Czapek broth medium, on a shaking incubator for 7 days at 30°C and 120 rpm. Forty ml of culture fluid was harvested through centrifugation at 5000 × g at 4°C for 15 min. The harvested pellet and supernatant were immediately stored at -70°C and later lyophilized. The lyophilized supernatant was mixed with 1 ml autoclaved distilled water. Surface sterilized seeds [55] were incubated overnight with Uniconazol (Sumitomo Heavy Chemical Co. Ltd., Takarazuka, Japan), 20 ppm (in autoclaved distilled water), to further minimize activity of seed coat gibberellins. The treated seeds were washed thoroughly and soaked in autoclaved distilled water until radical emergence. The young seedlings were transplanted in glass tubes containing 0.8% water-agar medium and grown in a growth chamber. A 10 μl of supernatant solution of fungal culture filtrate was applied on apical meristem of rice seedlings at two leaves stage. The shoot and plant length was observed after a week of culture filtrate application and compared with waito-c rice seedlings treated either with distilled water or culture filtrate of *G. fujikuroi*.

### Bioassay on *Atriplex gemelinii*

The surface sterilized seeds [55] of *Atriplex gemelinii *were treated with uniconazol and then soaked in autoclaved distilled water for germination. The two leaves stage seedlings were treated with culture filtrate (10 ul) of fungal strain IR-3-3. The shoot lengths were recorded after 15 days and compared with control treatments.

### Extraction and quantification of gibberellins

Fungal gibberellins (GAs) were extracted from culture filtrates after 7 days of incubation in Czapek broth according to an established protocol [56]. Extracted GAs were subjected to reverse-phase C18-HPLC. The GAs were chromatographed on a 3.9 × 300 mm μ Bondapak, C18 column (Waters Corp., Milford, MA, USA) and eluted at 1.5 ml min-1 with the following gradient: 0 to 5 min, isocratic 28% MeOH in 1% aqueous acetic acid; 5 to 35 min, linear gradient from 28 to 86% MeOH; 35 to 36 min, 86 to 100% MeOH; 36 to 40 min, isocratic 100% MeOH. Up to forty fractions of 1.5 ml each were collected. The fractions were then prepared for gas chromatograph/mass spectrometer (GC/MS) with selected ion monitoring (SIM) (6890N network GC system, and 5973 network mass selective detector; Agilent Technologies, Palo Alto, CA, USA). For each GA, 1 μl of sample was injected in a 30 m × 0.25 mm (i.d.), 0.25 μm film thickness DB-1 capillary column (J & W Scientific Co., Folsom, CA, USA). The GC oven temperature was programmed for a 1 min hold at 60°C, then to rise at 15°C min^-1 ^to 200°C followed by 5°C min^-1 ^to 285°C. Helium carrier gas was maintained at a head pressure of 30 kPa. The GC was directly interfaced to a Mass Selective Detector with an interface and source temperature of 280°C, an ionizing voltage of 70 eV and a dwell time of 100 ms. Full scan mode (the first trial), three major ions of the supplemented [^2^H_2_] GAs internal standards (obtained from Prof. Lewis N. Mander, Australian National University, Canberra, Australia) and the fungal gibberellins were monitored simultaneously. The retention time was determined using hydrocarbon standards to calculate the KRI (Kovats Retention Index) value, while the GAs quantification was based on peak area ratios of non-deuterated (extracted) GAs to deuterated GAs.

### Genomic DNA extraction and gel electrophoresis

An efficient method was developed for the isolation of genomic DNA from endophytic fungi, because usual CTAB extraction method and mycelial grinding was causing DNA shearing. Rich mycelial culture was obtained by growing fungus in Czapek culture broth (supplemented with 1% glucose and peptone) for 7 days on rotary shaking incubator (120 rpm and 28°C), and lyophilized for 24 hrs. A 0.5 g of lyophilized sample was broken carefully in 2 ml eppendorf, with blunt end spatula or glass rod. Double volume of lysis buffer (20 mM Tris-HCL, pH8.0; 10 mM EDTA; 1% SDS) containing 1% of 2- mercaptoethanol was added. The mixture was vortexed briefly (30 sec) to obtain homogeneity and left to incubate for 2 hr in water bath set at 55°C. 250 μl/ml of pre-heated 4% CTAB extraction buffer was added to lysed cells mixture and incubated further at 65°C for 1 hr. Chloroform extraction followed by iso-propanol precipitation yielded condensed strand of nucleic acid, which was cleaned from RNA using 10 ul of RNase A for 2 hr of incubation at 37°C. The isolated DNA was suspended in 50 ul of autoclaved deionized distilled water and tested for purity and quantity by agarose gel electrophoresis.

### PCR and identification

Fungal isolate was identified by sequencing internal transcribed region (ITS) of 18S rDNA, using universal primers ITS-1 (5'-TCC GTA GGT GAA CCT GCG G-3') and ITS-4 (5'-TCC TCC GCT TAT TGA TAT GC-3'). A 25 μl of PCR mixture contained 2.5 μl of dNTPs and Ex-Taq buffer, 2 μl of each primer, 0.5 μl of DNA sample, and 0.2 μl of Ex-Taq polymerase. Rest volume was adjusted with 15.3 μl of autoclaved deionized distilled water. The reaction cycle consisted of 2 min of initial denaturation at 95°C, followed by 35 cycles of 30 sec denaturation time (95°C), 60 sec of annealing (55°C) and 30 sec of extension (72°C), and a final extension time of 5 min at 72°C. The resultant product was gene cleaned using Nucleogen gene clean kit, ligated in T-vector using Takara Perfect T-cloning kit, and then inserted into DHα *E. coli *mutant cells (RBC) by overnight incubation (37°C). Plasmids with inserts were extracted using SolGent Plasmid mini-prep kit, and sequenced.

### Phylogenetic analysis

The BLAST search program  was used to look for nucleotide sequence homology for 18S ITS (1/4) region for fungi. The obtained sequences were aligned by ClustalW using MEGA version 4.0 software [57], and the neighbor-joining tree was generated using same software. Bootstrap replication (1000 replications) was used for a statistical support for the nodes in the phylogenetic tree.

## Authors' contributions

SAK and MH participated equally in overall experimentation and manuscript preparation. JGK, WSK and BML participated in design of the study. YSC and UHY participated in sampling area selection, plant collection and identification. SJS, SKH and JMK helped in root screening for fungal isolation. MH, HYK and IJL participated in gibberellins extraction and quantification. HY and JGK helped in gene isolation and identification. All authors read and approved the final manuscript.

## Supplementary Material

Additional file 1 **GC-MS SIM spectra for GA_1 _in culture filtrate of fungal isolate IR-3-3**. Arrow indicates the peak of fungal GA_1 _that coincides with that of internal standard GA_1_. Click here for file

Additional file 2 **GC-MS SIM spectra for GA**_3_**in culture filtrate of fungal isolate IR-3-3**. Arrow indicates the peak of fungal GA3 that coincides with that of internal standard GA3. Click here for file

Additional file 3 **GC-MS SIM spectra for GA**_4_**in culture filtrate of fungal isolate IR-3-3**. Arrow indicates the peak of fungal GA_4 _that coincides with that of internal standard GA_4_. Click here for file

Additional file 4 **GC-MS SIM spectra for GA**_7_**in culture filtrate of fungal isolate IR-3-3**. Arrow indicates the peak of fungal GA_7 _that coincides with that of internal standard GA_7_. Click here for file

Additional file 5 **GC-MS SIM spectra for GA_5 _in culture filtrate of fungal isolate IR-3-3**. Arrow indicates the peak of fungal GA_5 _that coincides with that of internal standard GA_5_. Click here for file

Additional file 6 **GC-MS SIM spectra for GA_5 _in culture filtrate of wild type *G. fujikuroi***. Arrow indicates the peak of fungal GA_5 _that coincides with that of internal standard GA_5_. Click here for file
