# Supplementary figures and images for: Plant growth promotion and Penicillium citrinum
Source: BMC Microbiol. 2008 Dec 22;8:231. doi: 10.1186/1471-2180-8-231 (PMC2631606; doi:10.1186/1471-2180-8-231)

Abundance

Ion 506.00 (505.70 to 506.70): K.P-528.D

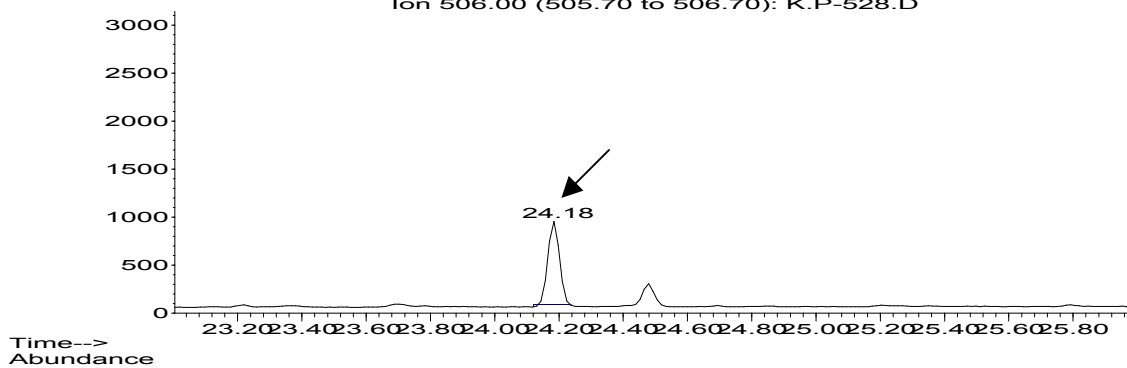

Time-->

Ion 508.00 (507.70 to 508.70): K.P-528.D

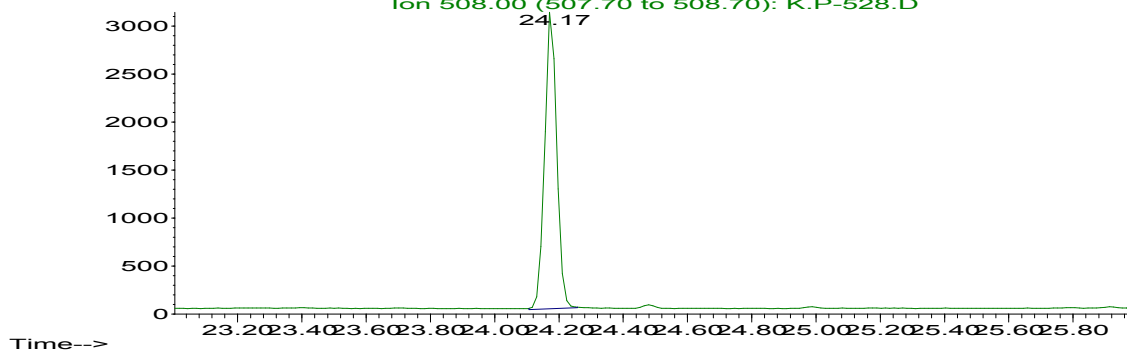

Time-->

Abundance

Scan 456 (24.184 min): K.P-528.D

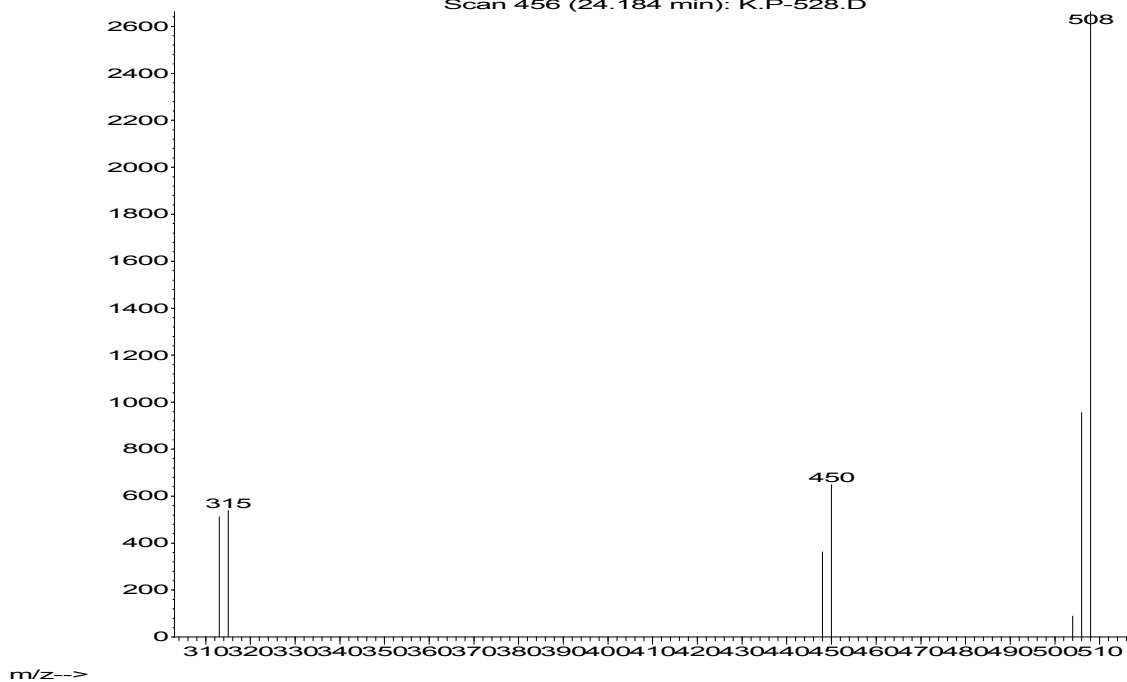

m/z-->

Supplement: Additional file 1 — GC-MS SIM spectra for GA1 in culture filtrate of fungal isolate IR-3-3. Arrow indicates the peak of fungal GA1 that coincides with that of internal standard GA1. [file 1471-2180-8-231-S1.pdf]

Abundance

Ion 504.00 (503.70 to 504.70): J.P-744.D

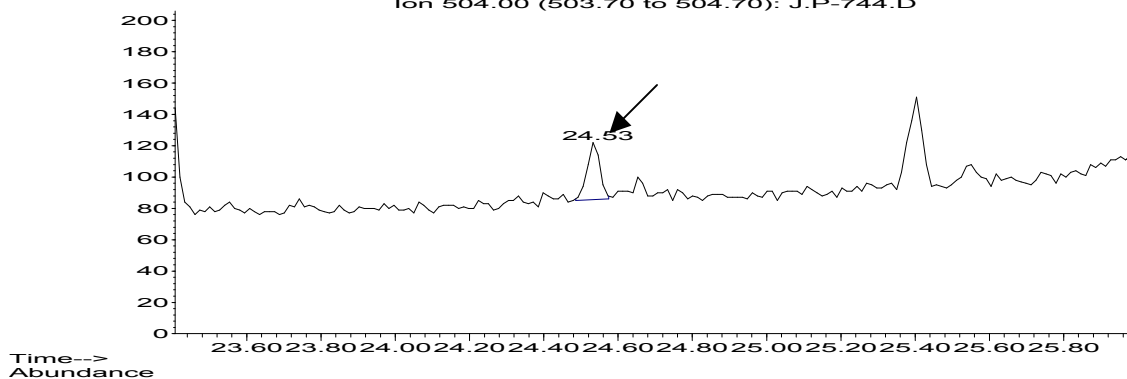

Time-->  
Abundance

Ion 506.00 (505.70 to 506.70): J.P-744.D

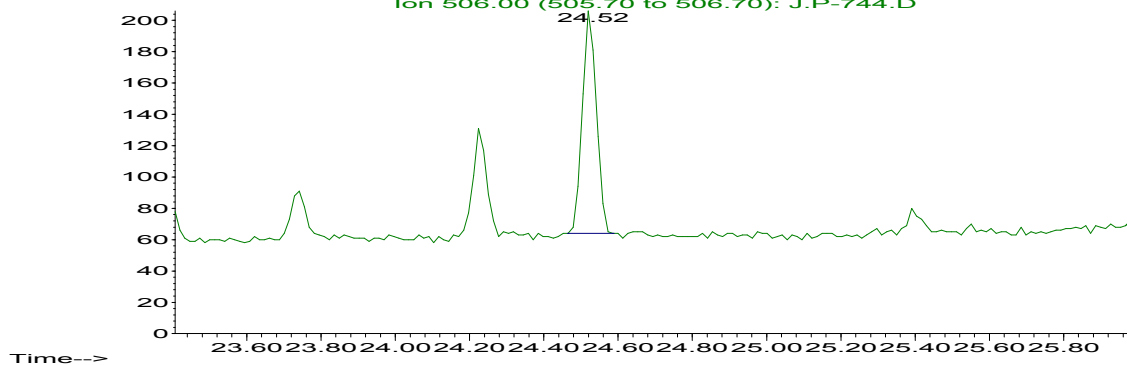

Time-->

Abundance

Scan 481 (24.519 min): J.P-744.D

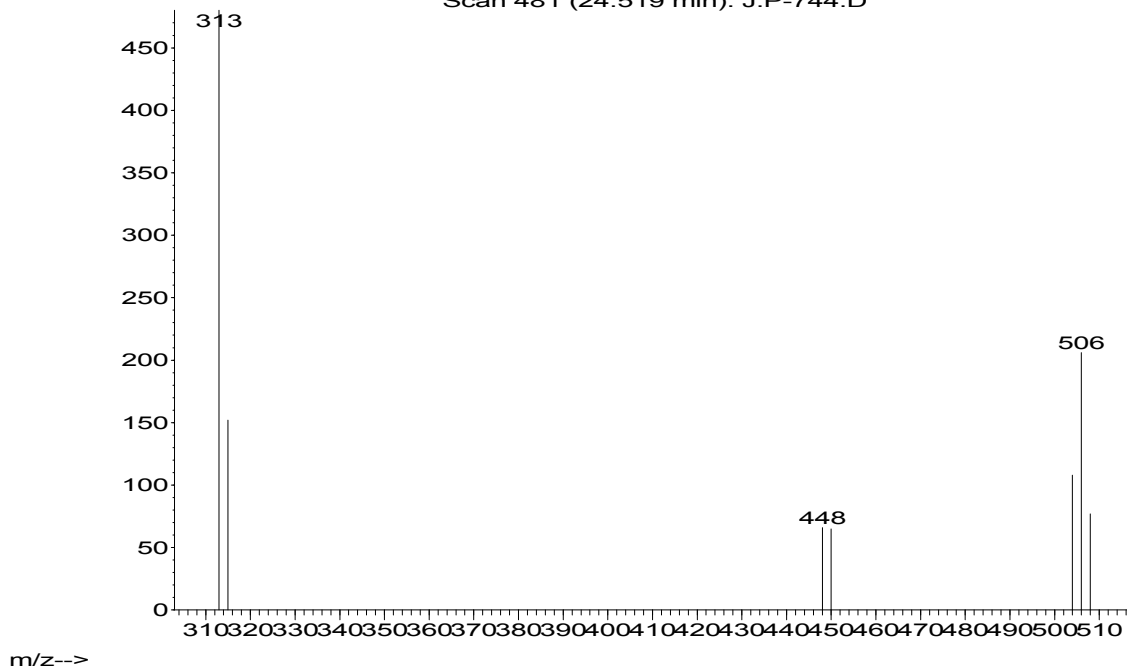

Supplement: Additional file 2 — GC-MS SIM spectra for GA3in culture filtrate of fungal isolate IR-3-3. Arrow indicates the peak of fungal GA3 that coincides with that of internal standard GA3. [file 1471-2180-8-231-S2.pdf]

Abundance

Ion 284.00 (283.70 to 284.70): J.P-906.D

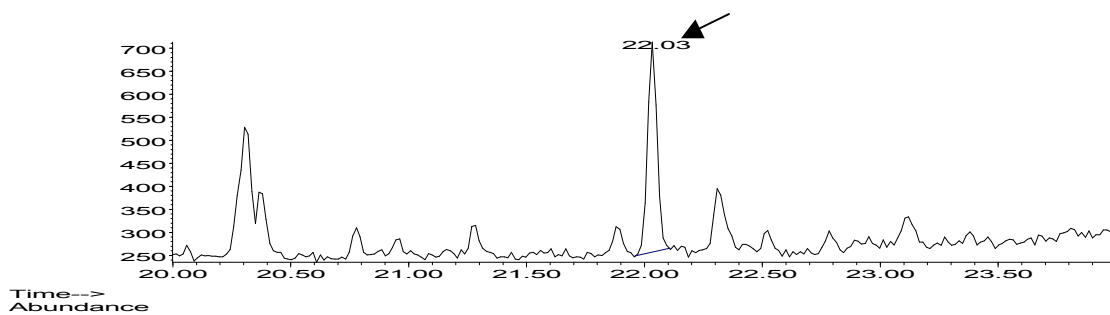

Ion 286.00 (285.70 to 286.70): J.P-906.D

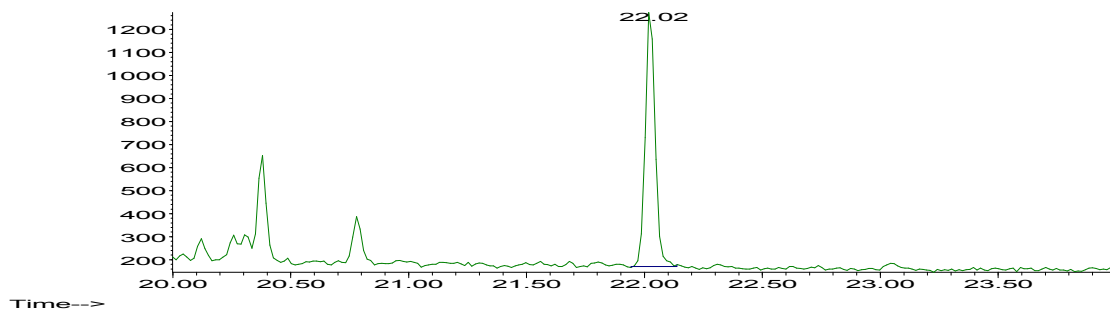

Abundance

Scan 259 (22.033 min): J.P-906.D

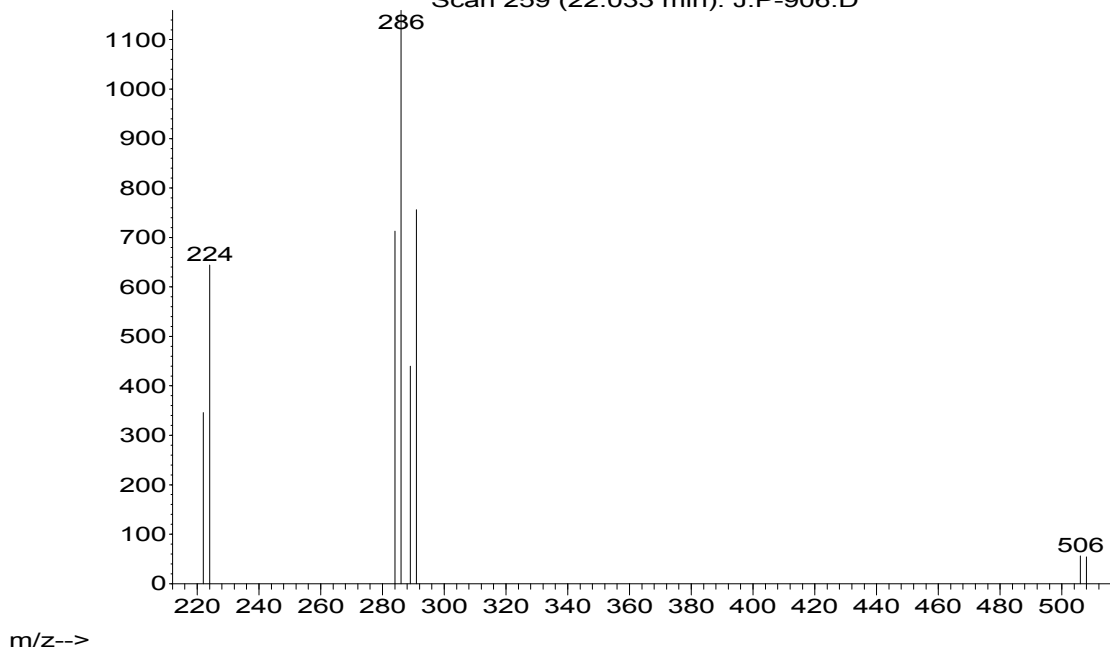

Supplement: Additional file 3 — GC-MS SIM spectra for GA4in culture filtrate of fungal isolate IR-3-3. Arrow indicates the peak of fungal GA4 that coincides with that of internal standard GA4. [file 1471-2180-8-231-S3.pdf]

Abundance

Ion 222.00 (221.70 to 222.70): J.P-906.D

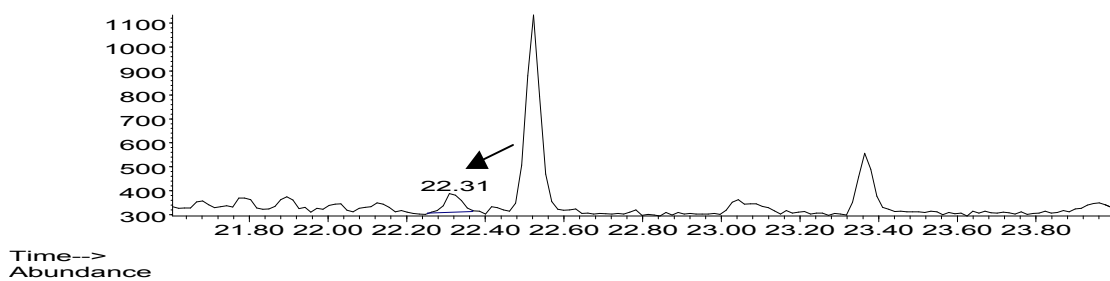

Ion 224.00 (223.70 to 224.70): J.P-906.D

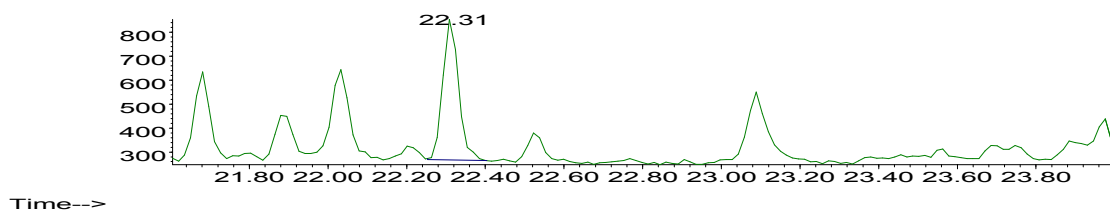

Abundance

Scan 277 (22.309 min): J.P-906.D

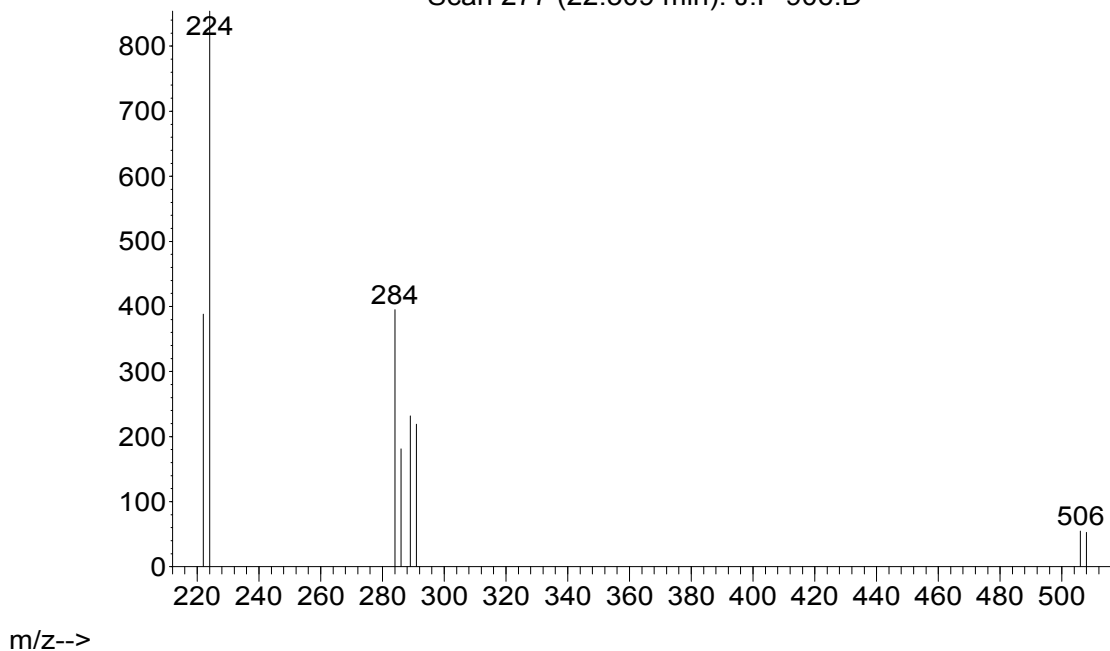

Supplement: Additional file 4 — GC-MS SIM spectra for GA7in culture filtrate of fungal isolate IR-3-3. Arrow indicates the peak of fungal GA7 that coincides with that of internal standard GA7. [file 1471-2180-8-231-S4.pdf]

Abundance

Ion 416.00 (415.70 to 416.70): K.P-077.D

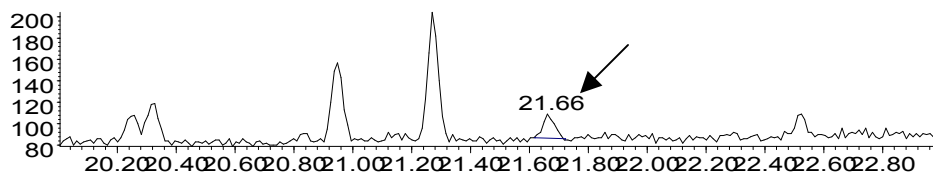

Time-->

Abundance

Ion 418.00 (417.70 to 418.70): K.P-077.D

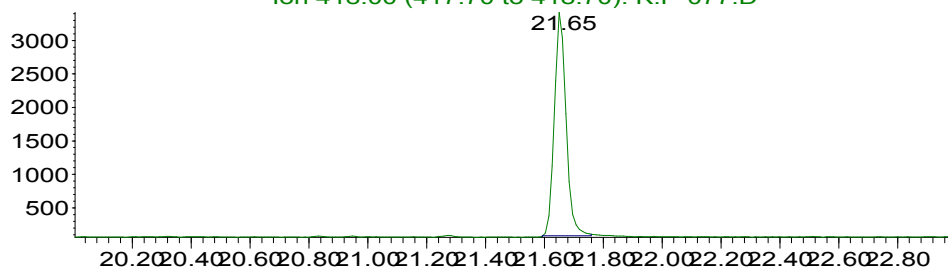

Time-->

Abundance

Scan 225 (21.661 min): K.P-077.D

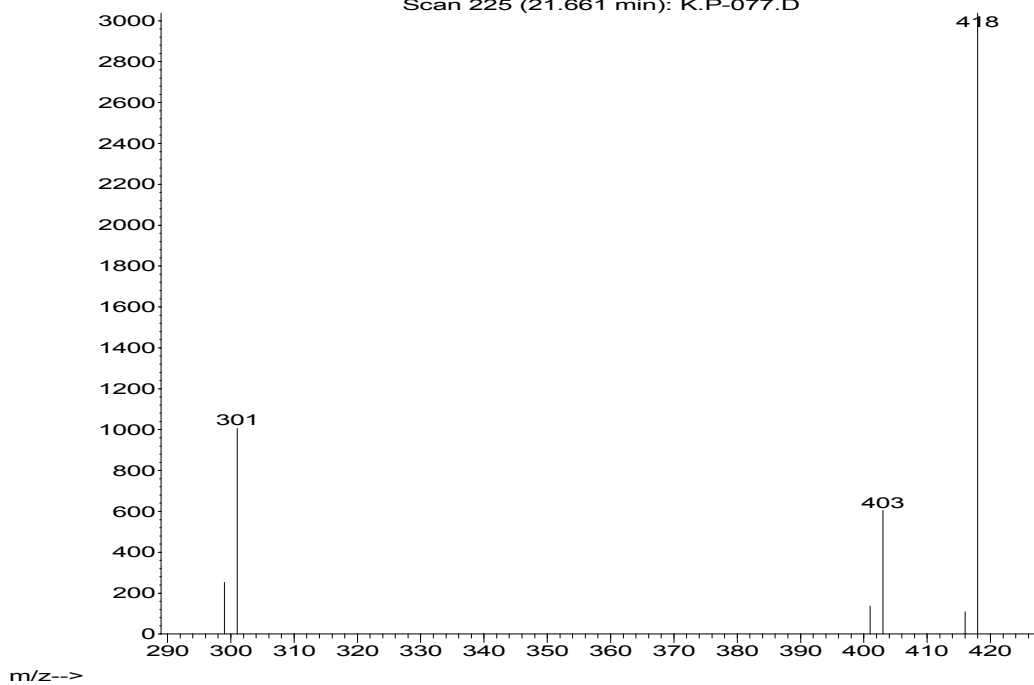

Supplement: Additional file 5 — GC-MS SIM spectra for GA5 in culture filtrate of fungal isolate IR-3-3. Arrow indicates the peak of fungal GA5 that coincides with that of internal standard GA5. [file 1471-2180-8-231-S5.pdf]

Abundance

Ion 416.00 (415.70 to 416.70): K.P-078.D

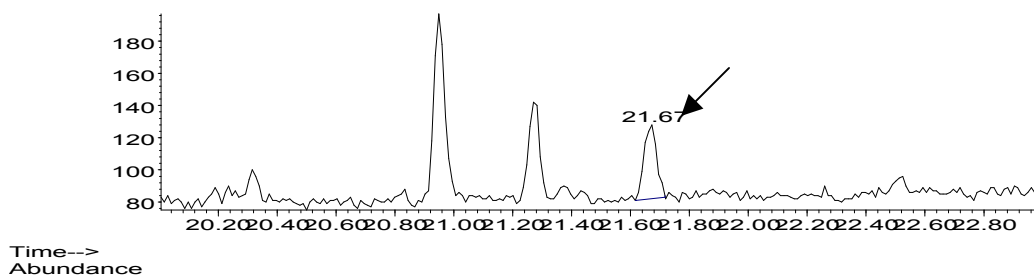

Ion 418.00 (417.70 to 418.70): K.P-078.D

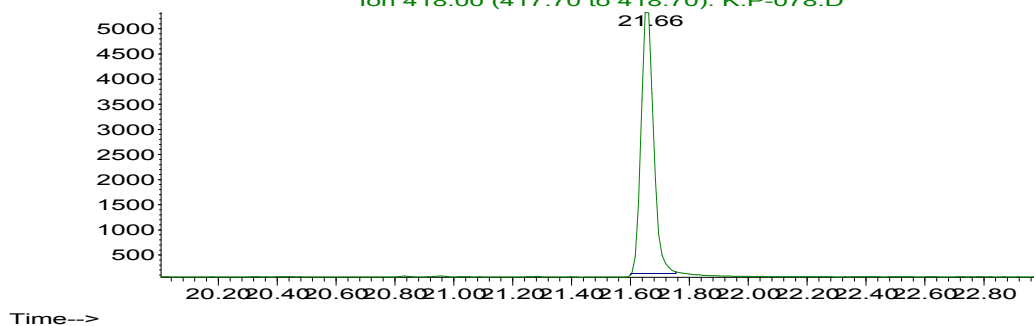

Abundance

Scan 225 (21.661 min): K.P-078.D

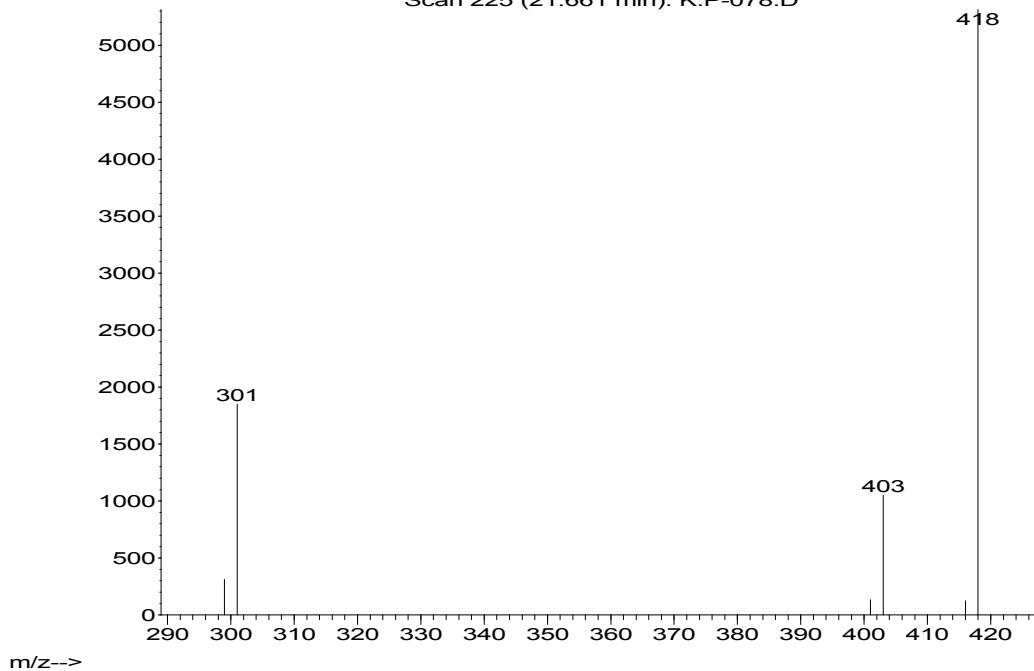

Supplement: Additional file 6 — GC-MS SIM spectra for GA5 in culture filtrate of wild type G. fujikuroi. Arrow indicates the peak of fungal GA5 that coincides with that of internal standard GA5. [file 1471-2180-8-231-S6.pdf]
